# Association between EEG metrics and continuous cerebrovascular autoregulation assessment: a scoping review

**DOI:** 10.1016/j.bja.2024.03.021

**Published:** 2024-04-20

**Authors:** Stefan Y. Bögli, Marina S. Cherchi, Erta Beqiri, Peter Smielewski

**Affiliations:** 1 Brain Physics Laboratory, Division of Neurosurgery, Department of Clinical Neurosciences, University of Cambridge, Cambridge, UK; 2 Department of Critical Care, Marqués de Valdecilla University Hospital, and Biomedical Research Institute (IDIVAL), Santander, Cantabria, Spain

**Keywords:** anaesthesia, cerebrovascular autoregulation, electroencephalography, multimodality neuromonitoring, neurocritical care

## Abstract

**Objective:**

Cerebrovascular autoregulation is defined as the capacity of cerebral blood vessels to maintain stable cerebral blood flow despite changing blood pressure. It is assessed using the pressure reactivity index (the correlation coefficient between mean arterial blood pressure and intracranial pressure). The objective of this scoping review is to describe the existing evidence concerning the association of EEG and cerebrovascular autoregulation in order to identify key concepts and detect gaps in the current knowledge.

**Methods:**

Embase, MEDLINE, SCOPUS, and Web of Science were searched considering articles between their inception up to September 2023. Inclusion criteria were human (paediatric and adult) and animal studies describing correlations between continuous EEG and cerebrovascular autoregulation assessments.

**Results:**

Ten studies describing 481 human subjects (67% adult, 59% critically ill) were identified. Seven studies assessed qualitative (e.g. seizures, epileptiform potentials) and five evaluated quantitative (e.g. bispectral index, alpha-delta ratio) EEG metrics. Cerebrovascular autoregulation was evaluated based on intracranial pressure, transcranial Doppler, or near infrared spectroscopy. Specific combinations of cerebrovascular autoregulation and EEG metrics were evaluated by a maximum of two studies. Seizures, highly malignant patterns or burst suppression, alpha peak frequency, and bispectral index were associated with cerebrovascular autoregulation. The other metrics showed either no or inconsistent associations.

**Conclusion:**

There is a paucity of studies evaluating the link between EEG and cerebrovascular autoregulation. The studies identified included a variety of EEG and cerebrovascular autoregulation acquisition methods, age groups, and diseases allowing for few overarching conclusions. However, the preliminary evidence for the presence of an association between EEG metrics and cerebrovascular autoregulation prompts further in-depth investigations.

**Editor's key points**

• Cerebrovascular autoregulation stabilises cerebral blood flow (CBF) during changing perfusion pressure. Electroencephalography (EEG) reflects changes in CBF. Possible interdependencies between EEG and cerebrovascular autoregulation have not previously been systematically assessed.
• The authors identified that seizures and burst suppression coincide with impaired cerebrovascular autoregulation. Alpha peak frequency and bispectral index reflect changes in cerebrovascular autoregulation.
• Few studies have investigated EEG and cerebrovascular autoregulation associations. Considering the strong temporal resolution of EEG, the assessment of dynamic time-domain features might permit deeper understanding of possible interdependencies.

Cerebrovascular autoregulation (CAR) is defined as the capacity of cerebral blood vessels to alter their resistance continuously to adapt to changing arterial blood pressure (or cerebral perfusion pressure [CPP]) and ultimately maintain a stable cerebral blood flow (CBF). Continuous monitoring of CAR has a prognostic value in acute brain injury and it can aid identifying individualised blood pressure targets within the limits of CAR.^1^^,^^2^ Different methods for CAR assessment are available. Most prominently, the pressure reactivity index (PRx: the correlation coefficient between 30 consecutive 10-s averages of arterial blood pressure [MAP] and intracranial pressure [ICP]) has been established as a standard for the continuous assessment of CAR in severe traumatic brain injury (TBI).^3^^,^^4^ PRx describes the association between changes in MAP and slow vasogenic changes of ICP.^1^ A positive correlation denotes impaired autoregulation with, in worst case, passive transmission of these slow waves to ICP. A negative corelation signifies active cerebrovascular response to changing MAP. If invasive monitoring using ICP is unavailable, the mean velocity index/mean velocity index arterial (Mx/Mxa: the correlation between slow waves in CPP/MAP and slow changes in CBF velocity measured using transcranial Doppler)^5^ or the cerebral oximetry index (COx: the correlation between MAP waves and changes in regional oxygen saturation measured using near infrared spectroscopy) can be calculated.^6^ CAR is dynamic and related to pathology severity and progression. While monitoring CAR is deemed important by the clinical community, the current metrics and indices suffer from lack of reliability and reproducibility.^7^

Electroencephalography (EEG) has a variety of clinical applications including diagnosis and monitoring of seizure and seizure-related disorders,^8^^,^^9^ monitoring of sedation, and detection of secondary complications.^10^, ^11^, ^12^ EEG can either be analysed visually to detect specific patterns (i.e. epileptiform potentials, seizures, and status epilepticus, burst suppression, etc.) or automatically using quantitative EEG (to assess power of specific frequency bands, spectral edge frequency, peak frequencies, etc.). There are various instances in which EEG reflects changes in CBF and vice versa. Increased neuronal activity (either of physiological or pathological origin such as seizures—assessed using EEG) leads to an increase of CBF as a result of an increased metabolic rate (i.e. increased oxygen and glucose consumption and increased waste removal).^13^, ^14^, ^15^ Hyperventilation leads to a generalised slowing of the EEG and decreased CBF.^16^^,^^17^ Decreases in CBF lead to distinct changes in EEG. The cortex is highly sensitive to ischemia and hypoxia because of its incessant need for uninterrupted oxygen and metabolite supply. During these decreases in CBF the EEG activity decreases too and is ultimately suppressed with the start of neuronal death.^18^^,^^19^ The changes occur within minutes after onset of ischemia and dynamically follow the changes in CBF. In addition to the association between EEG and CBF, EEG has been linked to changes in ICP. Burst suppression, for example, leads to a reduction in ICP.^20^ Other quantitative EEG metrics such as the power spectrum analysis derived pressure index,^21^ the slope of the power spectral density,^22^ or the bispectral index (BIS),^23^ have all been reported to reflect variations in ICP. Overall, an interdependence between EEG and CAR seems likely. Yet, the nature of these connections between specific EEG metrics or patterns and CAR indices remains obscure. Identification of such connections might ultimately improve understanding of CAR and potentially explain part of its dynamically changing nature and associated uncertainty.

## Review question

We performed a comprehensive scoping review to assess the currently available concepts, types of evidence, and individual results of studies evaluating the association between EEG metrics and continuous CAR assessment and to identify gaps in the current knowledge. A preliminary search of MEDLINE was conducted and no current or underway systematic reviews or scoping reviews on the topic were identified.

## Eligibility criteria

### Participants

This scoping review considered studies including healthy and ill human participants of both the paediatric and adult population and animal studies.

### Concept

The core concept studied in this scoping review is the association between continuous EEG parameters (both qualitative and quantitative) and continuous (>5 min) assessment of CAR.

### Context

This scoping review did not consider the specific sex/gender, geographic location, or race of participants.

## Types of sources

This scoping review considered both experimental and quasi-experimental study designs including randomised controlled trials, non-randomised controlled trials, before and after studies, and interrupted time-series studies. In addition, analytical observational studies including prospective and retrospective cohort studies, case-control studies, and analytical cross-sectional studies were considered for inclusion. This review also considered descriptive observational study designs and descriptive cross-sectional studies for inclusion. Text and opinion papers, and individual case reports were not considered for inclusion in this scoping review. Publications in languages other than English were not considered for inclusion.

## Methods

This scoping review was conducted in accordance with the Joanna Briggs Institute methodology for scoping reviews.^24^ The template provided by the Joanna Briggs Institute was used for drafting of this manuscript (https://jbi.global/scoping-review-network/resources). Reporting was performed in accordance with the Preferred Reporting Items for Systematic reviews and Meta-Analyses extension for Scoping Reviews (PRISMA-ScR) Checklist.^25^

### Search strategy

An initial limited search of MEDLINE was undertaken to identify articles on the topic. The text words contained in the titles and abstracts of relevant articles, and the index terms used to describe the articles were used to develop a full search strategy for Embase, MEDLINE, SCOPUS, and Web of Science. A detailed search strategy for MEDLINE, including all identified keywords and index terms can be found in Supplementary Appendix 1. The search strategy was adapted for each included database. The reference list of all included sources of evidence were screened for additional studies. Only studies published in English were included. Embase, MEDLINE, SCOPUS, and Web of Science were all searched from their inceptions to September 2023.

### Study and source of evidence selection

**Fig 1:**
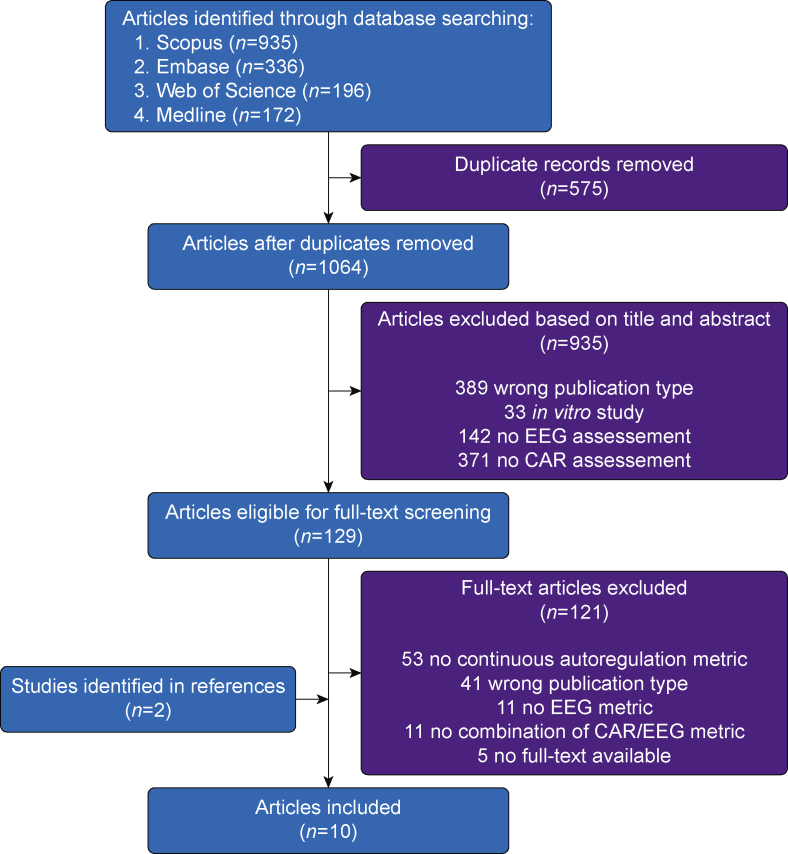
Review flow chart. CAR, Cerebrovascular autoregulation.

The study search and independent review were performed by SYB and MSC. A total of 1639 citations were identified, collated, and uploaded to Rayyan (https://rayyan.ai/). In a first step, Rayyan was used to identify possible duplicates, which were then evaluated by the reviewers comparing the title, abstract, DOI, and date of publication to assess whether these were actual duplicates. A total of 575 duplicates were removed. The residual titles and abstracts were screened independently for assessment against the inclusion criteria for this scoping review. After exclusion of 935 publications, a total of 129 full texts were assessed in detail against the inclusion criteria by the same independent reviewers. Any disagreements that arose between the reviewers at each stage of the selection process were resolved through discussion. A flow chart describing the process and the reasons for exclusion is presented in Figure 1.

### Data extraction

Data were extracted manually from papers included in the scoping review by two reviewers. The data extracted include specific details about the participants (study population, sample size), concept (EEG methodology, EEG duration, EEG metric/s, CAR metric/s, CAR monitoring duration, intervention), potential confounders (sedation), study methods (study design), and key findings relevant to the review question. Any disagreements that arose between the reviewers were resolved through discussion.

## Results

A total of 10 manuscripts were included in the final analysis. The characteristics of these studies can be found in Table 1. Overall, these studies included 481 patients (322 [67%] adults,^26^^,^^28^, ^29^, ^30^, ^31^, ^32^, ^33^ 159 [33%] children and adolescents^27^^,^^34^^,^^35^) with 117 (24%) TBI,^29^^,^^33^^,^^34^ 73 (15%) subarachnoid haemorrhage (SAH),^26^ 50 (10%) out-of-hospital cardiac arrest,^28^ 46 (10%) critically ill non-brain injured,^27^^,^^32^ and 195 (41%) non-critically ill elective or cardiac surgery patients.^30^^,^^31^^,^^35^ All the studies were observational and 80% were prospective. Sample sizes ranged from 12 to 80 patients per study. No animal studies could be identified.

**Table 1:** Study characteristics. BIS, bispectral index; BISopt, optimal bispectral index; CAR, cerebrovascular autoregulation; COx/COxa, cerebral oximetry index/cerebral oximetry index arterial; COPI, cerebral oximetry/pressure index; CPP, cerebral perfusion pressure; CPPopt, optimal cerebral perfusion pressure; deltaCPP, difference between actual CPP and CPPopt; EEG electroencephalogram; IIC, interictal continuum; LLA, lower limit of autoregulation; MAP, mean arterial blood pressure; Mx/Mxa, mean velocity index/mean velocity index arterial; PRx pressure reactivity index.

Author (year)	Aims/purpose	Study design	Population	Sample size	Key findings	Conclusion
Alkhachroum (2020)^26^	To evaluate whether impaired CAR, and non-optimal cerebral perfusion are associated with increased risk of seizures and IIC.	Retrospective observational	Adults; poor-grade subarachnoid haemorrhage	73	Patients with seizures/IIC have longer duration of supraoptimal CPP; deltaCPP/PRx increase 1 h before until the end of seizure/IIC.	Cerebral hyperaemia (supraoptimal CPP) is associated with seizures and IIC.
Chegondi (2023)^27^	To determine the effect of non-ictal EEG changes on CAR.	Prospective, observational	Children (0–18 yr); critically ill without acute brain injury, without electrographic of clinical seizures	16	There is no difference in number of abnormal 5-min EEG segments per 30 min epoch of preserved/impaired CAR.	Abnormal EEG findings are not associated with impaired CAR.
Crippa (2021)^28^	To investigate changes in CAR in patients treated with targeted temperature management after cardiac arrest.	Prospective, observational	Adults with out-of-hospital cardiac arrest undergoing targeted temperature management (32–34°C core temperature >24 h)	50	Patients with highly malignant patters have higher Mxa during normothermia.	Impaired CAR is associated with highly malignant patterns during normothermia.
Froese (2022)^29^	To assess whether an optimal level of sedation (BISopt) can be achieved using BIS and COxa.	Retrospective observational	Adults with moderate-to-severe traumatic brain injury	42	There is a U-shaped curve relationship between BIS and COxa.	Both light and deep sedation are associated with impaired CAR.
Liu (2021)^30^	To assess whether BIS is associated with cerebral perfusion during cardiopulmonary bypass during constant anaesthetic concentration.	Prospective, observational	Adults receiving isolated or combined cardiac artery bypass graft, valve, aortic or myectomy surgery	79	BIS values are lower during periods with MAP below the LLA *vs* above the LLA.	BIS is associated with MAP below the LLA in patients with constant anaesthetic administration.
Manquat (2022)^31^	To evaluate whether EEG can be used as a proxy measure for loss of CAR.	Prospective, observational	Adults requiring elective interventional neuroradiology procedures requiring general anaesthesia	36	Alpha peak frequency is independently (corrected for age, ASA class, norepinephrine infusion rate) associated with impaired CAR. No difference in band power.	Assessment of CAR during general anaesthesia based on EEG (which is routinely monitored) might be feasible.
Schramm (2012)^32^	To investigate the status of CAR within 24 h after the first signs of sepsis.	Prospective, observational	Adults; severe sepsis or septic shock	30	There was no correlation between CAR and the presence of EEG-based diagnosis of sepsis-related delirium.	Impaired CAR is associated with clinical but not EEG based diagnosis of sepsis-related delirium.
Thorat (2008)^33^	To explore the effects of barbiturate coma on cerebral tissue oxygen tension and PRx in patients with refractory intracranial hypertension.	Prospective observational	Adults; severe traumatic brain injury undergoing barbiturate infusion to achieve burst suppression	12	Barbiturate coma leads to decrease of PRx in survivors, but not in non-survivors compared with pre-barbiturate coma.	Barbiturate induced burst suppression is associated with an improvement of CAR in survivors.
Xie (2023)^34^	To investigate whether early EEG features predict ICP, CAR, brain tissue oxygenation, and functional outcome.	Prospective observational	Children and adolescents (aged <21 yr) with traumatic brain injury	63	Increased suppression percentage, alpha-delta ratio, delta power, and complexity are associated with increased PRx.	Increased suppression percentage on initial EEG recordings may predict impaired CAR.
Zou (2023)^35^	To evaluate the frequency of disturbed CAR in children with congenital heart disease early after cardiac surgery.	Prospective observational	Children; congenital heart disease undergoing cardiac surgery	80	COPI associated with background abnormalities, seizure duration, number of spikes/sharp waves, intraoperative isoelectric state, and abnormal background by 48 h. Duration of impaired COPI associated with seizure duration, number of delta brushes, abnormal sleep-wake cycling, intraoperative isoelectric state, and abnormal background by 48 h.	Magnitude and duration of impaired CAR is associated with different EEG markers.

### EEG: methods, durations, and metrics

Table 2 shows the characteristics of EEG and CAR assessment. The definitions of the EEG patterns and metrics described can be found in Table 3. Some 50% of the studies used 16-lead/10–20 EEG montages for acquisition,^27^^,^^28^^,^^32^^,^^34^^,^^35^ while 40% used reduced 2–8 channel EEG acquisition methods (e.g. bispectral index/BIS (Covidien, Minneapolis, MN, USA) monitoring, Masimo SedLine (Masimo, Irvine, CA, USA)).^29^, ^30^, ^31^^,^^33^ Only one study used intracortical EEG electrodes for continuous acquisition of EEG measurements.^26^ The exact duration of EEG was missing in most studies with the main description being ‘continuous’. Different qualitative and quantitative measures were evaluated in the different studies. Most studies focused on the evaluation of qualitative markers such as seizures (30% of studies), interictal epileptiform potentials or the ictal interictal continuum (IIC) (40% of studies) and highly malignant patterns/burst suppression (40% of studies).^26^, ^27^, ^28^^,^^32^, ^33^, ^34^, ^35^ Fewer studies analysed quantitative metrics such as BIS (20% of studies), power (and derivatives band power, alpha-delta ratio, asymmetry—20% of studies), and alpha peak frequency/complexity (each 10% of studies).^30^^,^^31^^,^^34^ Sedation was described in 80%^27^, ^28^, ^29^, ^30^, ^31^, ^32^, ^33^, ^34^ of studies and accounted for in 50%^27^^,^^29^, ^30^, ^31^^,^^33^ of studies.

**Table 2:** Characteristics: EEG/CAR monitoring. AtS, alpha-to-slow ratio; BIS, bispectral index; CAR, cerebrovascular autoregulation; COPI, cerebral oximetry/pressure index; COx/Coxa, cerebral oximetry index/cerebral oximetry index arterial; CPPopt, optimal cerebral perfusion pressure; deltaCPP, difference between actual CPP and CPPopt; EEG, electroencephalogram; IIC, interictal continuum; LLA, lower limit of autoregulation; Mx/Mxa, mean velocity index/mean velocity index arterial; PRx pressure reactivity index.

Author (year)	EEG methodology	EEG duration	EEG metric	CAR metric	CAR duration	Sedation described/analysed
Alkhachroum (2020)^26^	Intracortical EEG (1 in the skull, 2–3 in the cortical grey matter, 4–5 in the white matter)	Continuous (>48 h)	Ictal (>10 min), interictal continuum (IIC, >10 min), non-ictal	PRx, CPPopt, deltaCPP	Continuous (>48 h)	No/no
Chegondi (2023)^27^	16-Lead EEG	Continuous	Normal *vs* abnormal	COx	Continuous	Yes/yes
Crippa (2021)^28^	10–20 EEG	Continuous (>48 h)	Highly malignant pattern	Mxa	Once during/once after hypothermia	Yes/no
Froese (2022)^29^	Bilateral 4-channel BIS	Continuous	BIS	PRx, COxa	Continuous	Yes/yes
Liu (2021)^30^	Unilateral 2-channel BIS	Intraoperative	BIS	Mx, LLA	Intraoperative	Yes/yes
Manquat (2022)^31^	Bifrontal 4-channel EEG (Masimo)	Peri-interventional	Band power, total power, AtS, alpha peak frequency	Mxa	30 Min	Yes/yes
Schramm (2012)^32^	16-Lead 10–20 EEG	Once on day 4 after onset	EEG grading 1–4	Mx (30 consecutive 6-s averages)	60 Min/day	Yes/no
Thorat (2008)^33^	8-Lead EEG	Continuous	Burst suppression	PRx	Continuous	Yes/yes
Xie (2023)^34^	10–20 EEG	First 24 h of monitoring	Band power, alpha-delta ratio, complexity, asymmetry, suppression percentage, presence of seizures or interictal epileptiform potentials	PRx	Continuous (up to 7 days)	Yes/no
Zou (2023)^35^	10–20 EEG	48 h	Background category. Number of spike/sharp waves/hour. Seizure duration/hour. Abnormal sleep-wake cycling. Delta brushes	COPI (adapted COx: 50 consecutive 6-s averages)	Continuous	No/no

**Table 3:** Definitions of EEG and CAR metrics. AtS, alpha-to-slow ratio; BIS, bispectral index; CAR, cerebrovascular autoregulation; CBF, cerebral blood flow; COPI, cerebral oximetry/pressure index; COx/Coxa, cerebral oximetry index/cerebral oximetry index arterial; CPP, cerebral perfusion pressure; CPPopt, optimal cerebral perfusion pressure; deltaCPP, difference between actual CPP and CPPopt; EEG, electroencephalogram; ICP, intracranial pressure; IIC, interictal continuum; LLA, lower limit of autoregulation; MAP, mean arterial blood pressure; Mx/Mxa, mean velocity index/mean velocity index arterial; PRx, pressure reactivity index; rSO_2_, regional oxygen saturation.

CAR metric	CAR definition	EEG: Qualitative metrics	EEG: Qualitative metrics definitions	EEG: Quantitative metric	EEG: Quantitative metric definitions
COx/COxa/COPI^6^	Pearson correlation coefficient of 30 consecutive 10-s MAP/rSO_2_ averages	Abnormal sleep-wake cycling^36^	Sleep–wake cycling absence in neonates or absence of stage 2 sleep transients in children	Alpha-delta ratio	Ratio of alpha and delta power
CPPopt^37^	CPP at the lowest PRx value identified by a parabolic curve fitted to 5-min median CPP and PRx values, with bins of CPP of 5 mm Hg	Burst suppression^38^	Alternating pattern of attenuation/suppression and high voltage activity (>50% attenuation/suppression)	Alpha peak frequency	Frequency with maximal power within alpha band
deltaCPP^37^	True CPP–CPPopt	Background category^36^	Background (normal, mild, moderate, severe)	Asymmetry^39^	Percent absolute asymmetry within frequency bandwidth between hemispheres
LLA^30^	MAP value at which Mx is >0.4 with decreasing MAP	Delta brushes^38^	0.3–1.5 Hz activity superimposed with fast 8–22 Hz activity	AtS	Alpha–slow power
Mx/Mxa^5^	Pearson correlation coefficient of 30 consecutive 10-s CPP or MAP/CBF averages.	EEG grading 1–4^40^	1: Theta-delta rhythm, 2: delta rhythm, 3: triphasic waveforms, 4: burst suppression	BIS	Bispectral index
PRx^1^	Pearson correlation coefficient of 30 consecutive 10-s ICP/MAP averages	Epileptiform potentials	Spike/sharp waves/sharp and slow wave complexes (≥2.5 times background voltage, <200 ms)	Complexity^41^	Hjorth complexity parameter
		Highly malignant pattern^42^	Suppression (<10 μV) or burst suppression at any time	Power spectral density (PSD)	Periodogram calculated using the Welch method
		Ictal (seizures)^38^	Epileptiform potentials lasting for ≥10 s at ≥3 Hz or with evolution (frequency/morphology/location) at ≥1 Hz	Suppression percentage	Relative duration of >0.5-s epochs with <3 μV EEG activity
		Interictal continuum (IIC)^38^	Same as ictal but without evolution	Total/band power	Average PSD of defined frequency band (slow 0.1–1 Hz, delta 1–4 Hz, theta 4–8 Hz, alpha 8–14 Hz, beta 13–20 Hz, total 0.1–25 Hz)
		Normal *vs* abnormal	Normal (physiological/sedation) *vs* abnormal (burst suppression, periodic lateral epileptiform discharges, non-ictal increased activity)		

### Cerebrovascular autoregulation: methods, durations, and metrics

Cerebrovascular autoregulation was evaluated with time-domain correlation coefficients methods, based on transcranial Doppler (Mx/Mxa—40% of studies),^28^^,^^30^, ^31^, ^32^ ICP (PRx/optimal CPP [CPPopt]—40% of studies),^26^^,^^29^^,^^33^^,^^34^ or near infrared spectroscopy (NIRS) (COx, cerebral oximetry index arterial [COxa], cerebral oximetry/pressure index [COPI]—30% of studies).^27^^,^^29^^,^^35^ The CAR indices and calculation methods used are described in Table 3. NIRS- and ICP-based metrics were evaluated continuously, while Mx/Mxa were evaluated intermittently with recording lengths of 30–60 min. The exact duration of CAR evaluation was missing in some studies (described as continuous).

### Associations between EEG and cerebrovascular autoregulation

Alkhachroum and colleagues^26^ evaluated a cohort of 73 poor grade SAH patients. PRx, CPPopt, and deltaCPP (difference between actual CPP and CPPopt) were assessed comparing patients with seizures or IIC to patients without either. Interestingly, PRx and CPPopt increased starting from 1 h before the onset of seizure or IIC on EEG. Furthermore, CPP was higher in patients with seizures/IIC leading to an overall increased positive deltaCPP compared to patients without seizures/IIC. However, when considering overall, per-patient, averages of PRx/CPPopt, no difference could be found between patients with and without seizures/IIC.

Chegondi and colleagues^27^ assessed critically ill non-brain-injured children comparing qualitative EEG metrics (burst suppression, periodic lateral epileptiform discharges, non-ictal increased activity) with increased COx (cut-off 0.2–0.4). They found no difference in the number of consecutive 5-min segments with impaired CAR within 30 min of normal/abnormal EEG.

Crippa and colleagues^28^ assessed 50 patients undergoing targeted temperature management after out-of-hospital cardiac arrest comparing the presence of highly malignant patterns with Mxa during and after hypothermia. Overall, patients with highly malignant patterns in normothermia had higher Mxa values describing impaired autoregulation.

Froese and colleagues^29^ described a new method of optimising sedation based on autoregulation termed BISopt in a cohort of 42 adults with TBI. They found a U-shaped relationship between BIS and COxa showing that both oversedation and undersedation carry the risk of impairing CAR.

Liu and colleagues^30^ evaluated 79 patients undergoing cardiopulmonary bypass during cardiac surgery comparing BIS to Mxa based estimation of the lower limit of autoregulation. BIS (during stable anaesthetic administration) was lower when MAP fell below the lower limit of autoregulation with a linear relationship between BIS and extent of MAP below the lower limit of autoregulation.

Manquat and colleagues^31^ evaluated 36 patients undergoing non-emergency neuroradiology surgery evaluating different quantitative EEG metrics (band power, total power, alpha minus slow power, alpha peak frequency) against CAR evaluated using Mxa. Impaired CAR was independently associated with lower alpha peak frequency. None of the other metrics showed a difference between intact and impaired CAR.

Schramm and colleagues^32^ investigated 30 patients with severe sepsis or septic shock describing the presence of specific encephalopathy/delirium associated patterns compared with Mx. No association was found.

Thorat and colleagues^33^ described 12 patients undergoing barbiturate infusion to achieve burst suppression for the treatment of refractory intracranial hypertension. Burst suppression led to an improvement of CAR evaluated using PRx in survivors, but not in patients who ultimately died.

Xie and colleagues^34^ evaluated 63 children and adolescents with TBI comparing different qualitative (suppression percentage, seizures, interictal epileptiform potentials) and quantitative (band power, alpha-delta ratio, complexity, asymmetry) EEG metrics to PRx. Increased suppression percentage, alpha-delta ratio, delta power, and complexity were associated with increased PRx.

Zou and colleagues^35^ investigated 80 children with congenital heart disease undergoing cardiac surgery. The described different qualitative EEG characteristics (background category, number of epileptiform potentials per hour, seizure duration per hour, presence of abnormal sleep-wake cycling, delta brushes) compared against COPI. Increased COPI was associated with background abnormalities, seizure duration, number of spikes/sharp waves, intraoperative isoelectric state, and abnormal background. Furthermore, duration of impaired COPI was associated with seizure duration, number of delta brushes, abnormal sleep-wake cycling, and abnormal background activity.

A summary of the associations found between the different CAR metrics and the qualitative/quantitative measures of EEG can be found in Table 4. Table 5 summarises the number of studies evaluating each combination of EEG/CAR metric and the consistency of results (either correlated, inconsistent, or not correlated). Overall, few combinations were evaluated by two studies (PRx *vs* seizures/IIC^26^^,^^34^; COx/COxa/COPI *vs* IIC and interictal epileptiform potentials,^27^^,^^35^ PRx *vs* burst suppression^33^^,^^34^) with all other combinations being evaluated by maximally one study.

**Table 4:** Combined results comparing EEG and CAR metrics. AtS, alpha-to-slow ratio; BIS, bispectral index; CAR, cerebrovascular autoregulation; COPI, cerebral oximetry/pressure index; COx/Coxa, cerebral oximetry index/cerebral oximetry index arterial; CPPopt, optimal cerebral perfusion pressure; deltaCPP, difference between actual CPP and CPPopt; EEG, electroencephalogram; IIC, interictal continuum; LLA, lower limit of autoregulation; MAP, mean arterial blood pressure; Mx/Mxa, mean velocity index/mean velocity index arterial; PRx, pressure reactivity index.

	Seizures	IIC/interictal epileptiform discharges	Encephalopathy/background abnormalities	Highly malignant pattern/burst suppression (induced or spontaneous)	BIS	Power (band/total/alpha delta ratio/asymmetry)	Peak frequency	Other
**PRx**	PRx increases 1 h before seizure^26^; no difference between seizure/control group^26^; no association^34^	PRx increases 1 h before IIC^26^; no difference between IIC/control group^26^; no association^34^		Barbiturate coma decreases PRx in survivors, but not in non-survivors^33^; suppression % associated with PRx^34^		No association with asymmetry, theta/alpha/beta band power^34^; association between delta band power/alpha delta ratio^34^		No association with complexity^34^
**deltaCPP/CPPopt**	Higher deltaCPP in seizure group^26^	Higher deltaCPP/CPPopt in IIC group^26^						
**COx/COxa/COPI**	Seizure duration associated with worse CAR^35^	Number of epileptiform discharges associated with worse CAR^35^; no association between number of 5-min impaired CAR chunks and abnormal 30-min EEG^27^	Background abnormalities associated with worse CAR^35^	No association between number of 5-min impaired CAR chunks and abnormal 30-min EEG^27^	There is a U-shaped association between BIS and COxa^29^			No association with abnormal sleep/wake cycling and delta brushes^35^; intraoperative isoelectric state associated with worse CAR^35^
**Mx/Mxa**			No association to delirium EEG^32^	Highly malignant pattern associated with worse CAR during normothermia after CA^28^	Linear relationship between MAP and BIS below LLA^30^	No association between alpha/delta/total and AtS *vs* CAR^31^	Alpha peak frequency associated with Mxa^31^

**Table 5:** Frequency of evaluation and consistency of associations found. ADR, Alpha-delta ratio; BIS, bispectral index; COPI, cerebral oximetry/pressure index; COx/Coxa, cerebral oximetry index/cerebral oximetry index arterial; CPPopt, optimal cerebral perfusion pressure; deltaCPP, difference between actual CPP and CPPopt; IIC, interictal continuum; Mxa, mean velocity index arterial; PRx, pressure reactivity index.

Frequency of evaluation (consistency)	Seizures	IIC/interictal epileptiform discharges	Encephalopathy/background abnormalities	Highly malignant pattern/burst suppression (induced or spontaneous)	BIS	Power (band/total/ADR/asymmetry)	Peak frequency	Other
PRx	2 (Inconsistent)	2 (Inconsistent)		2 (Correlation)		1 (Inconsistent)		1 (No Correlation)
deltaCPP/CPPopt	1 (Correlation)	1 (Correlation)						
COx/COXa/COPI	1 (Correlation)	2 (Inconsistent)	1 (Correlation)	1 (No correlation)	1 (Correlation)			1 (Inconsistent)
Mxa			1 (No correlation)	1 (Correlation)	1 (Correlation)	1 (No correlation)	1 (Correlation)	

## Discussion

This scoping review shows the paucity of data regarding possible association between EEG markers and metrics of CAR. However, certain promising links have been reported and are worth investigating further. The most prominent relationship reported comprises the association between seizures and impaired autoregulation.^26^^,^^35^ Within patients who eventually suffered from seizures or displayed an IIC, PRx already increased 1 h before onset.^26^ Furthermore, longer seizures were tied to worse CAR.^35^ There exist compelling explanations for the concomitance of seizures and CAR failure. Seizures lead to an increase in oxygen consumption and metabolic rate.^11^^,^^43^ To compensate, CBF is increased by means of vasodilation thus potentially leading to saturation of CAR capacity,^44^^,^^45^ an effect commonly seen in severe hypercapnia. In fact, Alkhachroum and colleagues^26^ described the tie-in between the seizure occurrence and increase of end-tidal CO_2_. It must be noted that rapid changes in carbon dioxide partial pressure (*P*aco_2_) levels violate the assumptions of PRx, which should only be calculated when a steady state of ventilation has been achieved, thus making PRx values unreliable for those periods.^46^ It is unclear, how long it takes for *P*aco_2_ to stabilise after onset/cessation of seizures. Seizures also lead to changes in intracranial or intrathoracic pressure by means of vasodilation,^47^ impairment of ventilation, pulmonary oedema,^48^^,^^49^ impairment of the blood brain barrier,^50^^,^^51^ metabolic crisis,^43^ or systemic inflammatory response.^52^^,^^53^ These changes can also affect CAR directly (e.g. inflammatory response/ventilation), or indirectly through resulting intracranial hypertension. The description of changes in CAR up to 1 h before the onset of seizures and IIC are more challenging to explain.^26^ On the one hand, impaired CAR might lead to hyperaemia which the authors describe as a possible risk factor for seizures, and on the other hand, because of the limited resolution of EEG, the seizure might have started in other regions, already affecting CAR, before propagating.

Burst suppression describes a distinct EEG pattern which is described most commonly as a consequence of severe hypoxic brain injury (such as after out-of-hospital cardiac arrest)^54^ but can also occur after severe brain injury other than cardiac arrest.^55^ Unlike seizures, burst suppression and electroclinical silence are not linked to an increased, but decreased metabolic load.^56^, ^57^, ^58^ Induction of burst suppression can be used as a therapeutic measure for refractory intracranial hypertension, severe vasospasm, or status epilepticus. Highly malignant patterns after out-of-hospital cardiac arrest and increased suppression percentage after paediatric TBI were associated with worse CAR likely as a representation of the severity of brain injury (i.e. extent of ischemia).^28^^,^^34^ The effect of burst suppression on CAR directly remains unclear.^20^

Overall, quantitative measures of EEG were examined less often. There are plausible associations between BIS as a measure of sedation and CAR.^29^ Insufficient sedation in intensive care patients leads to increased levels of stress/agitation,^59^ difficulties in ventilation,^60^ and increased ICP.^61^ All these changes alter cerebral perfusion and might lead to impaired CAR. Oversedation could lead to a direct impairment of vascular reactivity^29^^,^^62^ and reduction of CBF.^63^ Furthermore, with MAP decreasing below the lower limit of autoregulation, occurrence of hypoperfusion and ischemia is more likely, and this leads to slowing of EEG activity and thus lower BIS values.^30^ Power-based metrics have only been evaluated in two studies with inconsistent results.^31^^,^^34^ One factor that might have led to these inconsistent results is the effect of brain trauma on the EEG, which itself causes alterations and most commonly reduction of the EEG activity potentially concealing associations with CAR.^64^

### Limitations

The main limitation within the literature is the low number of studies covering various age groups, diseases, and most importantly measuring methods/evaluated metrics. Few studies evaluated potential confounders of changing CAR such as sedation and ventilation. Sedation can alter various EEG metrics. Burst suppression occurs as a consequence of the severity of disease or represents the desired effect of a therapeutic intervention. Most, if not all, of the patients received pharmacological sedation as part of the treatment regimen. Yet, clear descriptions comparing sections before and after either induced or spontaneous burst suppression are described in only one study^33^ compromising inference. Results were described as overall averages or covering prespecified time points (i.e. day 1, day 2, etc. or pre-/post-seizures). A higher granularity and potentially a within-subject design might reveal further, currently concealed interdependences.

Considering the limitations of this scoping review, certain studies might have been missed because of the exclusion of manuscripts published in languages other than English and as databases other than Medline, Embase, Scopus, and Web of Science were not searched. Furthermore, as a result of the dynamic and changing nature of CAR, we decided to focus on the evaluation of continuous measures of CAR excluding imaging-based, static methods of CAR estimation such as xenon-computer tomography and perfusion weighted imaging which might have provided information on the overall association between CAR and EEG metrics.

### Future directions

The studies identified provide preliminary evidence concerning aspects of the relationship and interactions between CAR and EEG metrics. Various gaps in the current knowledge remain. Because of the sparse number of studies with little methodological overlap, validation studies are necessary. In addition, there are some specific areas where clear knowledge gaps exist that require further, in depth, investigation. In particular, the vascular reactivity, as is evaluated using PRx, is a highly dynamic process and so are the processes represented collectively by the EEG metrics. Evaluation of dynamic time-domain features would potentially allow for deeper understanding of possible interdependences between the two. For example, brain function, and corresponding EEG metrics, are dependent on intact CAR upholding adequate blood supply. In patients with impaired CAR, it would be of clinical interest to know whether certain EEG metrics can ascertain the extent of resulting hypoperfusion and whether manipulation of CPP, which in absence of a CAR would translate to direct manipulation of CBF, could still be beneficial for the patient. Along the same lines, it is currently unclear whether targeting CAR might even reduce the number of seizures occurring at times as a consequence of hypoperfusion or hyperperfusion. A specific aspect of interest would be evaluating the difference between spontaneous and induced burst suppression allowing for the differentiation between a neuroprotective measure and a representation of the severity of disease. EEG metrics such as burst suppression can be indicative of the extent of the neuronal damage which is linked to CAR failure. Increased neuronal activity (for instance as a result of seizures) might excessively increase the metabolic load rendering the concurrent CAR strength ineffective, thus supporting the use of burst suppression as a therapeutic measure. To answer those specific questions, meticulously annotated multimodal neuromonitoring data would be necessary. Such data may in fact already be available in some centres, given the steadily increasing interest in full resolution, waveform level, monitoring data acquisition coupled with technological advances, and increased availability, of electronic medical records systems, automatically recording detailed drug delivery information.

### Conclusions

In this systematically performed scoping review we identified a total of 10 studies evaluating the relationship between continuous assessment of EEG and cerebrovascular autoregulation. The different studies identified used various EEG and cerebrovascular autoregulation acquisition methods and metrics, across both paediatric/adult patient populations, with different diseases, limiting the possibility of overarching conclusions. However, they also elucidate some preliminary evidence for the presence of an association between specific EEG metrics and cerebrovascular autoregulation, prompting further in-depth investigations and potentially animal experiments.

## Authors’ contributions

Conceived the study: SYB, PS

Contributed to the study design: all authors

Data collection and analysis: SYB, MSC

Interpretation of the results: all authors

Wrote the first draft of the manuscript: SYB

Commented on and revised the manuscript, and approved the final manuscript: all authors

## Declaration of interest

The authors declare that they have no conflicts of interest.

## Funding

The Swiss National Science Foundation (210839 to SYB). The Cantabrian Health Service (López Albo Post-Residency Program PESI/1/22 to MSC). EB is supported by the Medical Research Council (MR N013433-1) and by the Gates Cambridge Scholarship.
