# Supplementary figures and images for: Association between EEG metrics and continuous cerebrovascular autoregulation assessment: a scoping review
Source: Br J Anaesth. 2024 Apr 20;133(3):550–64. doi: 10.1016/j.bja.2024.03.021 (PMC11347808; doi:10.1016/j.bja.2024.03.021)

Appendix 1. Search Strategy MEDLINE


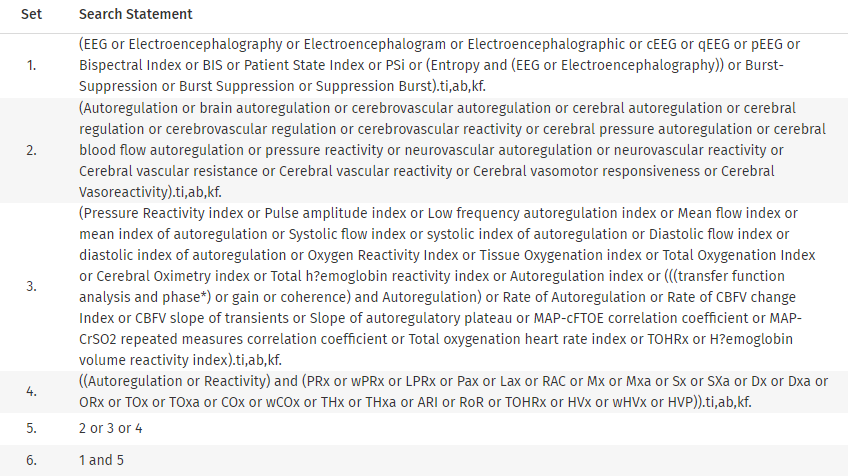

Supplement: Multimedia component 1 [file mmc1.docx]
